# Development and characterization of an oral multispecies biofilm implant flow chamber model

**DOI:** 10.1371/journal.pone.0196967

**Published:** 2018-05-17

**Authors:** Nadine Kommerein, Katharina Doll, Nico S. Stumpp, Meike Stiesch

**Affiliations:** Clinic for Dental Prosthetics and Biomedical Materials Science, Hannover Medical School, Hannover, Germany; Oregon Health & Science University, UNITED STATES

## Abstract

Peri-implant infections are the most common cause of implant failure in modern dental implantology. These are caused by the formation of biofilms on the implant surface and consist of oral commensal and pathogenic bacteria, which harm adjacent soft and hard tissues and may ultimately lead to implant loss. In order to improve the clinical situation, there has to be a better understanding of biofilm formation on abiotic surfaces. Therefore, we successfully developed a system to cultivate an oral multispecies biofilm model in a flow chamber system, optimized for the evaluation of biofilm formation on solid materials by direct microscopic investigation. The model contains four relevant oral bacterial species: *Streptococcus oralis*, *Actinomyces naeslundii*, *Veillonella dispar* and *Porphyromonas gingivalis* in ratios similar to the native situation. The reliability of the developed “Hanoverian Oral Multispecies Biofilm Implant Flow Chamber” (HOBIC) model was verified. Biofilm volume and live/dead distribution within biofilms were determined by fluorescence staining and confocal laser scanning microcopy (CLSM). The individual species distribution was analyzed using quantitative real time PCR with propidium monoazide pretreatment (PMA-qRT-PCR) and by urea-NaCl fluorescence *in situ* hybridization (urea-NaCl-FISH). This *in vitro* model may be used to analyze biofilm formation on dental implants in more detail and to develop future implant systems with improved material properties.

## Introduction

Due to demographic changes and increased life expectancy, the demand for biomedical products will increase steadily in the future. Their largest proportion by far is claimed by dental materials, with dental prostheses accounting for approximately 50% of them [1]. More than 1.2 million dental implants are currently inserted annually in Europe and this number is expected to increase [2]. However, dental implants are also among the implanted medical devices that suffer from the highest rate of implant-associated infection [3]. In the 5–10 years after implantation, peri-implantitis develops in up to 10% of implants and 20% of patients [4]. These infections are caused by bacterial biofilms [3,5–7]. Biofilms are defined as microbial communities that are irreversibly attached to a substratum that is surrounded by a self-produced matrix of extracellular polymeric substances (EPS) and where the microbial phenotype is modified with respect to growth rate and gene transcription compared to the planktonic counterparts [7].

Ca. 700 bacterial species have already been identified in the human mouth, with up to 500 species in an individual oral cavity [8,9]. These bacteria build a highly structured oral multispecies biofilm. The initial colonizers of this biofilm are streptococci, actinomyces and veillonellae [10–12]. Streptococci and actinomyces are able to co-aggregate, bind to molecules adsorbed on a surface and provide binding sites for the attachment of further bacteria [13–15]. Veillonellae form metabolic relationships with streptococci and are able to link early and late colonizing bacteria [13,15]. All of these bacteria are members of the commensal biofilm community at healthy sites of the oral cavity [16]. If their biomass increases excessively due to poor oral hygiene, parodontopathogenes like the anaerobic *Porphyromonas gingivalis* are attracted by increasing levels of intergeneric signaling molecules [14,17,18]. These are thought to misdirect the host immune response and increase proinflammatory response [12,19–23]. The bacterial composition shifts from a commensal to a pathogenic community accompanied by gingival detachment and crestal bone loss—referred to as peri-implantitis -, which can finally lead to implant failure. Retrospective treatment of mature biofilms is difficult due to inherent resistance mechanisms. The surrounding EPS matrix acts as a diffusion barrier and drastically reduces antibiotic penetration and host phagocytosis [24–28]. Furthermore, bacteria inside the biofilm exhibit reduced growth rates and unique gene expression patterns, thereby bypassing the point of attack of common antibiotics [24,29–31]. As a consequence, bacteria organized in a biofilm may be up to 1000-fold less susceptible to antibiotic treatment than planktonic cultures [24].

To combat biofilm-related implant infections and their consequences for patients and the health care system, *in vitro* models are needed to investigate oral biofilm formation on implant materials in order to develop novel materials, which inhibit biofilm formation from the early beginning. To mimic the environment in the oral cavity, such models should include an oral multispecies biofilm, physiological flow conditions and the implant material. There are already several flow cell systems for the cultivation of oral monospecies biofilms on implant/orthodontic materials [32–36], as well as oral multispecies biofilm models grown under static [37–40] or flow conditions [41–43]. In contrast, the number of test systems comprising all three components is limited. Astasov-Frauenhoffer et al. [44–47] developed and characterized a three-species biofilm model, composed of the initial colonizer *Streptococcus sanguinis*, the bridging bacterium *Fusobacterium nucleatum* and *P*. *gingivalis*, grown on the common implant material titanium in a flow chamber system. According to the relative species distribution, this model corresponds to a pathogenic oral biofilm. Blanc et al. [48] developed a six species oral biofilm cultivated in a flow chamber system on the implant material hydroxyapatite, which was dominated by aforementioned initial colonizers and corresponds therefore more to a commensal oral biofilm. Even though they demonstrated an effect of antibacterial mouth rinses on the multispecies biofilm, the reproducibility of the model itself was not addressed in detail.

The aim of the present study was to develop a system to analyze the formation of an oral multispecies biofilm on implant materials under physiological flow conditions, and to demonstrate its reliability with respect to biofilm formation and species distribution. The study employed a flow chamber system optimized for the testing of implant materials and which permits direct microscopic investigation of biofilm formation [36]. This system was equipped with specimens of titanium, a commonly used implant material. As the initial biofilm, which forms on an implant material and is thereby target to antibiofilm materials, is dominated by oral commensals and peridontopathogens are only found in considerably lower amounts, the following four species were involved: *Streptococcus oralis*, *Actinomyces naeslundii*, *Veillonella dispar* and *Porphyromonas gingivalis*. The experimental reproducibility of biofilm formation was confirmed by confocal laser-scanning microscopy (CLSM; biovolume determination and live/dead distrubution), quantitative real-time PCR (qRT-PCR; relative species distribution) and urea-NaCl fluorescence *in situ* hybridization (Urea-NaCl-FISH; spatial species distribution).

## Materials & methods

### Bacterial strains and culture conditions

*Streptococcus oralis* (ATCC^®^ 9811™) was purchased from the American Type Culture Collection (ATCC, Manassas, USA). *Actinomyces naeslundii* (DSM 43013), *Veillonella dispar* (DSM 20735) and *Porphyromonas gingivalis* (DSM 20709) were acquired from the German Collection of Microorganisms and Cell Cultures (DSMZ, Braunschweig, Germany). Bacteria were stored at -80°C and routinely cultivated in brain heart infusion medium (BHI; Oxoid, Wesel, Germany), supplemented with 10 μg/ml vitamin K (Roth, Karlsruhe, Germany) under anaerobic conditions (80% N_2_, 10% H_2_, 10% CO_2_) at 37°C prior to experiments.

### Multispecies biofilm formation in the flow chamber system

The flow chamber system was previously developed by Rath et al. [36] as a recirculating system. It was modified into an open system, where bacteria flow from the bioreactor through the flow chamber to a waste bottle. Titanium discs (grade 4)—12 mm in diameter finished with 45 μm diamond abrasive polishing wheels—were used as test specimens and inserted into the flow chambers. The system was prepared for anaerobic cultivation as described previously [36]. Bacterial precultures were adjusted to an optical density at 600 nm of 0.5 in BHI/vitamin K. This corresponds approximately to 2x10^13^ CFU/ml for *S*. *oralis*, 2x10^10^ CFU/ml for *A*. *naeslundii*, 5x10^8^ CFU/ml for *V*. *dispar* and 1x10^9^ CFU/ml for *P*. *gingivalis*. Equal volumes of the precultures were mixed and added to the bioreactor containing BHI/vitamin K to a 1:180 dilution. Biofilms were grown for 24 h at 37°C at flow rate of 100 μl/min under anaerobic conditions and protected from light. Flow chambers were separated from the bioreactor and prepared for further analysis as described previously [36].

### Fluorescent staining and biofilm volume quantification

Biofilms were washed with Dulbecco’s Phosphate Buffered Saline (PBS; Biochrom GmbH, Berlin, Germany) at a flow rate of 150 μl/min for 20 min to remove unbound bacteria. Subsequently, they were stained for fluorescence using the LIVE/DEAD^®^ BacLight^TM^ Bacterial Viability Kit (Life Technologies, Darmstadt, Germany). SYTO^®^9 and propidium iodide were applied simultaneously as a 1:1000 dilution in PBS at a flow rate of 150 μl/min for 20 min. Finally, biofilms were fixed with 2.5% glutardialdehyde (Roth, Karlsruhe, Germany) in PBS under the same conditions. The biofilms were examined by CLSM (Leica TCS SP8, Leica Microsystems, Mannheim, Germany). SYTO^®^9 dye was excited at 488 nm and the emission was measured from 500–550 nm; propidium iodide dye was excited at 552 nm and the emission was measured from 675–750 nm. Experiments were conducted in three biological replicates, each consisting of three titanium specimens. For each specimen, five image stacks were taken with a z-step size of 5 μm. The Imaris x64 8.4 software package (Bitplane AG, Zurich, Switzerland) was used for 3D reconstructions, volume calculation and to quantify the viable (SYTO^®^9; green), dead (propidium iodide; red) and colocalized (SYTO^®^9 + propidium iodide; orange) parts of the biofilms from the image z-stacks. Colocalized fluorescence was defined as part of propidium iodide staining, as the dye was able to penetrate the membrane. As it did not completely remove SYTO^®^9, it was subtracted from SYTO^®^9 staining.

### PMA treatment, DNA isolation and qRT-PCR

To remove planktonic bacteria on the top of the biofilm and simultaneously to maintain culture conditions, the remaining bioreactor medium was centrifuged and the bacteria-free supernatant was used to wash biofilms for 40 min at a flow rate of 150 μl/min. The flow chamber devices were removed from the system and opened under sterile conditions. The biofilm-covered titanium specimens were transferred to PBS and bacteria were detached by flushing with a pipette and carefully scraping with a cell scraper (Sarstedt, Nürnbrecht, Germany). Experiments were conducted in three biological replicates, each consisting of three titanium specimens. In addition, three samples each were collected of the mixed planktonic precultures before bioreactor inoculation (0 hours), and planktonic cultures in the bioreactor after 4 and 24 hours. All samples were treated with propidium monoazide (PMA) to selectively examine viable bacteria only [40,49,50]. The protocol was performed as described by Kommerein et al. [40] except for planktonic samples, which were treated with a final PMA concentration of 120 μM (4 hour planktonic samples) and 240 μM (24 hour planktonic samples) instead of 100 μM (starting mixtures and biofilms). Bacterial DNA was isolated using the FastDNA^TM^ SPIN Kit for Soil (MP Biomedicals, Eschwege, Germany), according to the manufacturer’s instructions but followed by ethanol precipitation. qRT-PCR was performed using the iQ5 real time PCR detection system (Bio-Rad, Hercules, California, USA). Primer pairs (S1 Table), reaction components (S2 Table) and cycle conditions (S3 Table) are listed in the supporting information. Each qPCR was carried out in duplicate. The amount of genomic DNA of each bacterial species in the planktonic and biofilm samples was calculated in comparison to a standard curve. By dividing the amount of DNA by the theoretical genome weight per cell, the number of bacterial cells could be calculated (see S4 Table in the supporting information) [40,51].

### Fluorescence *in situ* hybridization

Biofilms were fixed by pumping 50% ethanol (J.T. Baker, Phillipsburg, New Jersey, USA) through the system with a flow rate of 150 μl/min for 40 min. After fixation, the flow chamber device was removed from the system, opened under sterile conditions and the biofilm-covered titanium specimen was taken out and air-dried. The FISH protocol was modified from Lawson et al. [52] as applied in Kommerein et al. [40]. In brief, biofilms were permeabilized in 100 μl of 1 μg/ml lysozyme for 10 min; the reaction was stopped using 100 μl ethanol absolut. Hybridization was then performed for 30 min using 50 μl hybridization buffer and 4 μl of each probe. The applied 16S rRNA probes (Eurogentec, Cologne, Germany) are listed in S5 Table in the supporting information. Stained biofilms were covered with PBS and examined by CLSM (Leica TCS SP8, Leica Microsystems, Mannheim, Germany). In the first sequence, ALEXA Fluor^®^405 signals were detected with a PMT detector using a 405 nm laser and an emission range of 413–477 nm, together with ALEXA Fluor^®^568 (PMT detector / 552 nm laser / 576–648 nm emission range). In the second sequence, ALEXA Fluor®488 signals (PMT detector / 488 nm laser / 509–576 nm) were detected together with ALEXA Fluor^®^647 (PMT detector / 638 nm laser / 648–777 nm emission range). Image stacks were acquired with a z-step size of 2 μm. The Imaris x64 8.4 software package (Bitplane AG) was used for image stack processing.

### Statistical analysis

Data were documented and analyzed using the GraphPad Prism 6.04 software (GraphPad Software, Inc., La Jolla, USA). Biofilm volume and live/dead distribution were analyzed for Gaussian distribution using the D’Agostino & Pearson omnibus normality test. According to the results, biofilm volume was analyzed for equivalence using the Kruskal-Wallis test with Dunn’s multiple comparison correction, whereas live/dead distribution was analyzed for equivalence using the ordinary one-way ANOVA with Tukey’s multiple comparison correction. Biofilm qRT-PCR results were analyzed for equivalence by two-way repeated measures ANOVA with Tukey’s multiple comparison correction. Data were defined as equivalent with a p-value > 0.1.

## Results

### Four species growth in the bioreactor

**Fig 1:**
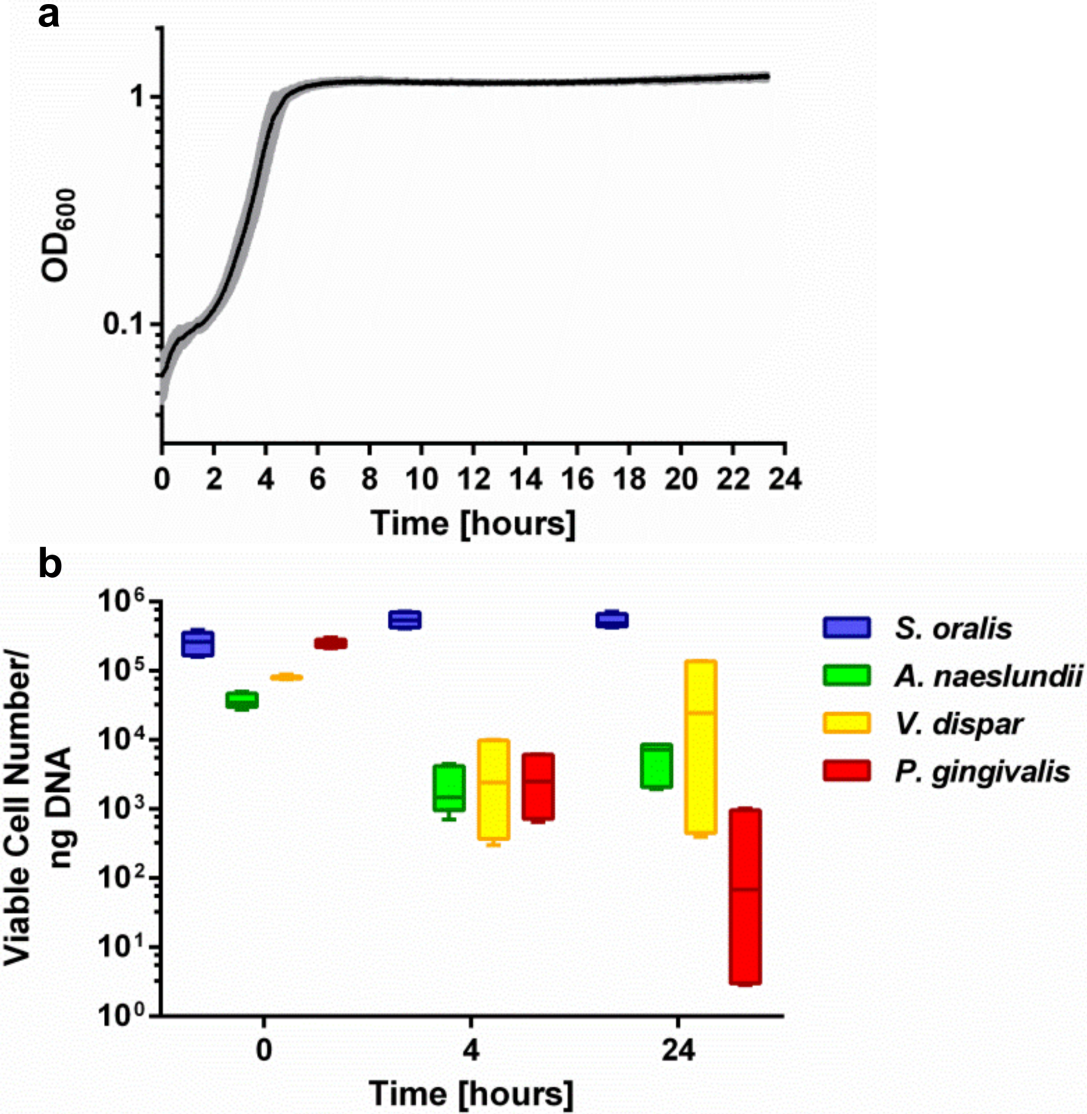
Four species growth in the bioreactor. (a) Mean ± standard deviation of the optical density at 600 nm (OD_600_) growth curve of *S*.*oralis*, *A*. *naeslundii*, *V*. *dispar* and *P*. *gingivalis* in the bioreactor for 24 hours. (b) Tukey box plots of viable species distribution in planktonic samples before inoculation (0 hours) and taken from the bioreactor after 4 and 24 hours analyzed by PMA-qRT-PCR.

*S*. *oralis*, *A*. *naeslundii*, *V*. *dispar* and *P*. *gingivalis* were simultaneously grown in a bioreactor, which feeds the flow chamber system. The optical density at 600 nm inside the bioreactor was continuously monitored using an inline photometer. The mean growth curve is depicted in Fig 1A. Initially, the optical density rises due to bacterial inoculation. After a lag phase of about 2 h, bacteria started exponential growth and reached the stationary phase after 6 h, with a final OD_600_ of approximately 1.2. At the beginning of the experiment (0 h), during the exponential growth phase (4 h), and at the end of the cultivation (24 h), planktonic samples were taken and examined by PMA-qRT-PCR for the distribution of viable bacteria. As shown in Fig 1B, viable cells of all species could be detected at all time points. The species with the highest abundance throughout all samples was *S*. *oralis*, followed by *V*. *dispar*, *A*. *naeslundii* and *P*. *gingivalis*, although the order changed over time of planktonic cultivation. The inoculum (0h) contained a mean of 2.7x10^5^ (± 9.2x10^4^) cells *S*. *oralis*, 2.5x10^5^ (± 3.7x10^4^) cells *P*. *gingivalis*, 8.1x10^4^ (± 4.4x10^3^) cells *V*. *dispar*, and 3.8x10^4^ (± 9.0x10^3^) cells *A*. *naeslundii* in 1 ng isolated DNA. After 4 h of cultivation in the bioreactor, the cell number of *S*. *oralis* slightly increased to 5.6x10^5^ (± 1.4x10^5^) per 1 ng isolated DNA, whereas the cell number of the other species decreased to 4.2x10^3^ (± 4.5x10^3^) for *V*. *dispar*, 2.2x10^3^ (± 1.7x10^3^) for *A*. *naeslundii* and 3.1x10^3^ (± 2.5x10^3^) for *P*. *gingivalis*. At the end of the experiment after 24 h, the amount of *S*. *oralis* was almost unchanged, with 5.3x10^5^ (± 1.2x10^5^) per 1 ng isolated DNA. The cell number of *V*. *dispar* and *A*. *naeslundii* increased to 5.4x10^4^ (± 6.6x10^4^) and 5.9x10^3^ (± 3.2x10^3^), respectively, whereas the amount of *P*. *gingivalis* further decreased to 3.5x10^2^ (± 4.9x10^2^) cells per 1 ng isolated DNA.

### Quantification of biofilm volume and live/dead distribution

**Fig 2:**
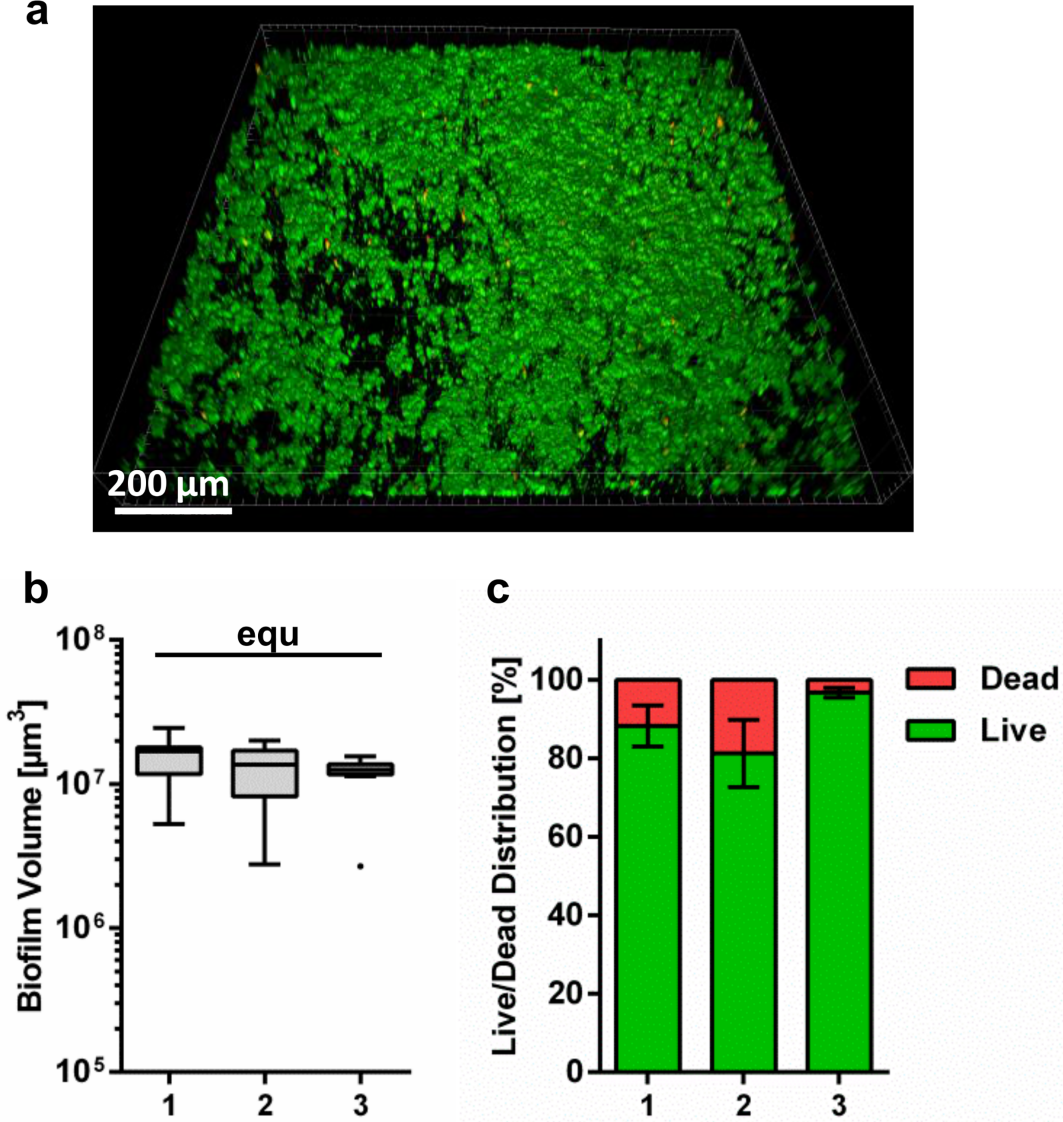
Quantification of biofilm volume and live/dead distribution. (a) 3D reconstruction of live/dead fluorescent stained 24 hours four species biofilm on titanium specimens grown in the flow chamber system. Viable bacteria are visualized in green and dead cells are visualized in red/orange. (b) Tukey box plots of the total biofilm volume per 1.2 x 1.2 mm^2^ and (c) mean ± standard deviation of live/dead distribution of total biofilm of three biological replicates after 24 h growth on titanium specimens in the flow chamber system. Statistically equivalent results are indicated with “equ”.

Biofilms were grown for 24 h in the flow chamber system on titanium specimens. They were subjected to live/dead fluorescent staining and analyzed by CLSM, in order to quantify biofilm volume and live/dead distribution. The formation of biofilm was observed on all titanium specimens. A representative image of the biofilm is shown in Fig 2A. The total biofilm volume of the three biological replicates (Fig 2B) was statistically equivalent. The average biofilm volume was 1.35x10^7^ μm^3^ (± 0.25x10^7^ μm^3^) per image of 1.2x1.2 mm^2^. For live/dead distribution, the biofilm on all titanium specimens appeared to be mostly viable (Fig 2A and 2C). The fraction of dead cells fluctuates between 5–19% in the different biological replicates (Fig 2C) and was not statistically equivalent. A mean of 87.3% (± 8%) of the biofilm was viable and 12.7% (± 8%) was dead.

### Quantification of the individual bacterial species within the biofilms

**Fig 3:**
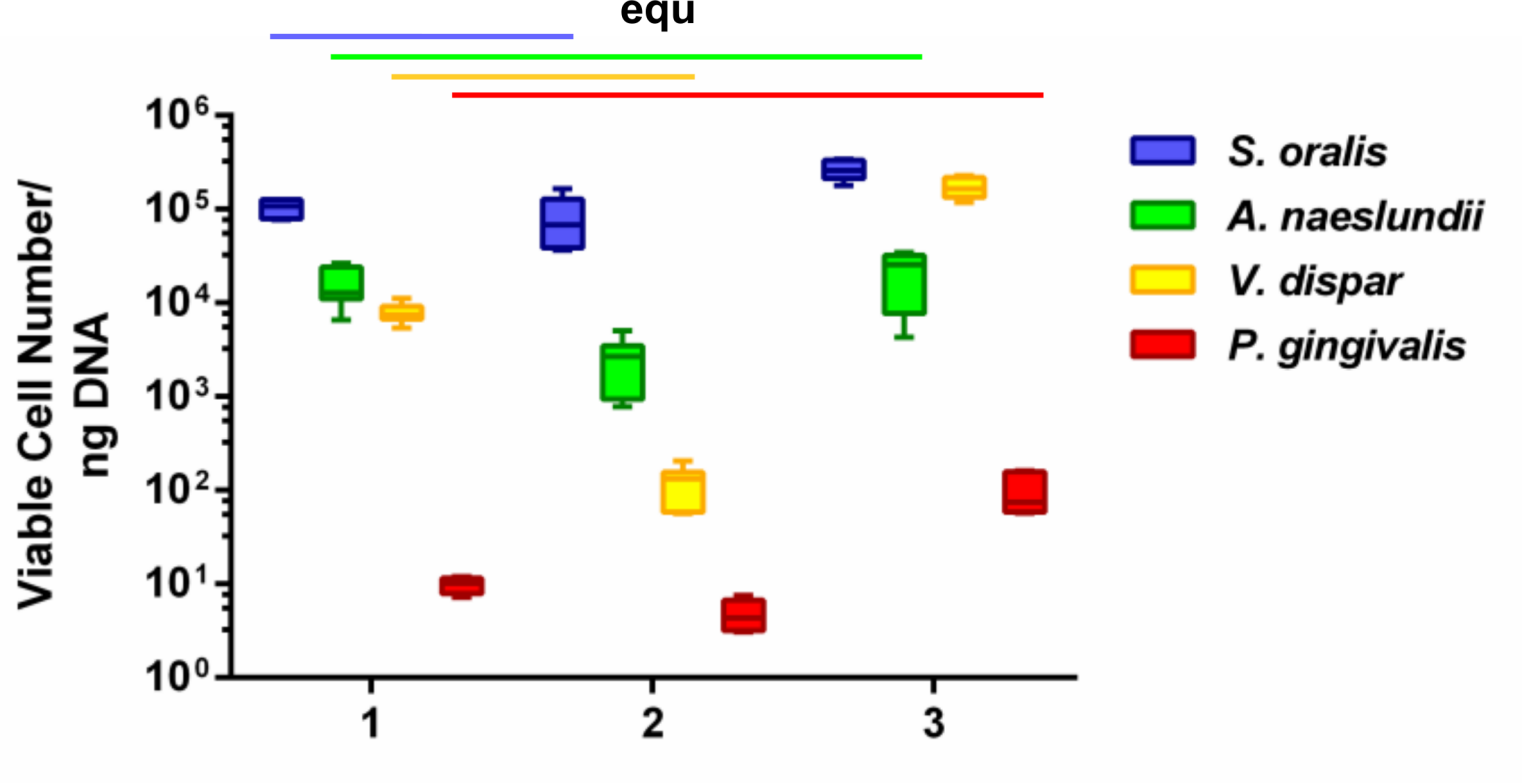
Viable species distribution within the 24 hour biofilms on titanium specimens in the flow chamber system. Tukey box plots of biofilm content of viable species distribution in three independent biological replicates (1.–3.) containing three technical replicates (three chambers) each, after 24 h growth on titanium specimens in the flow chamber system. PMA-qRT-PCR was run in duplicate for each biofilm sample. Statistically equivalent results are indicated with “equ”.

After 24 hours of growth, the biofilm samples were treated with PMA prior to DNA extraction—in order to analyze the viable cell count for each bacterial species in a subsequent qRT-PCR. PMA-qRT-PCR of three biological replicates containing three technical replicates revealed that all four bacterial species were integrated in each of the nine biofilms and that they were viable after 24 hours of co-cultivation (Fig 3). Within the three biological replicates, *S*. *oralis* was always the dominant species with (1.) 1.0x10^5^ (± 2.3x10^4^), (2.) 8.2x10^4^ (± 5.0 x10^4^), and (3.) 2.7x10^5^ (± 6.3x10^4^) cells/ng DNA. The lowest levels were found for *P*. *gingivalis* with (1.) 9.8 (± 1.9), (2.) 4.8 (± 1.8), and (3.) 97.2 (± 49.2) cells/ng DNA. Within the first two biological replicates, *A*. *naeslundii* was the second most abundant species with (1.) 1.6x10^4^ (± 7.6x10^3^) and (2) 2.5x10^3^ (± 1.5x10^3^) cells/ng DNA; *V*. *dispar* was the third most frequent species with (1.) 7.9x10^3^ (± 1.9x10^3^) and (2.) 1.2x10^2^ (± 5.6x10^1^) cells/ng DNA. The order was reversed in the third biological replicate; *V*. *dispar* was the second with (3.) 1.7x10^5^ (± 4.2x10^4^) and *A*. *naeslundii* the third most abundant species with (3.) 2.2x10^4^ (± 1.2x10^4^). Statistical comparison revealed that the counts of viable cells per ng DNA of *A*. *naeslundii* and *P*. *gingivalis* were equivalent within the three different biological replicates; *S*. *oralis* and *V*. *dispar* were statistically equivalent within the first two biological replicates (indicated with “equ” in Fig 3).

### FISH-based detection of the four species within the biofilm

**Fig 4:**
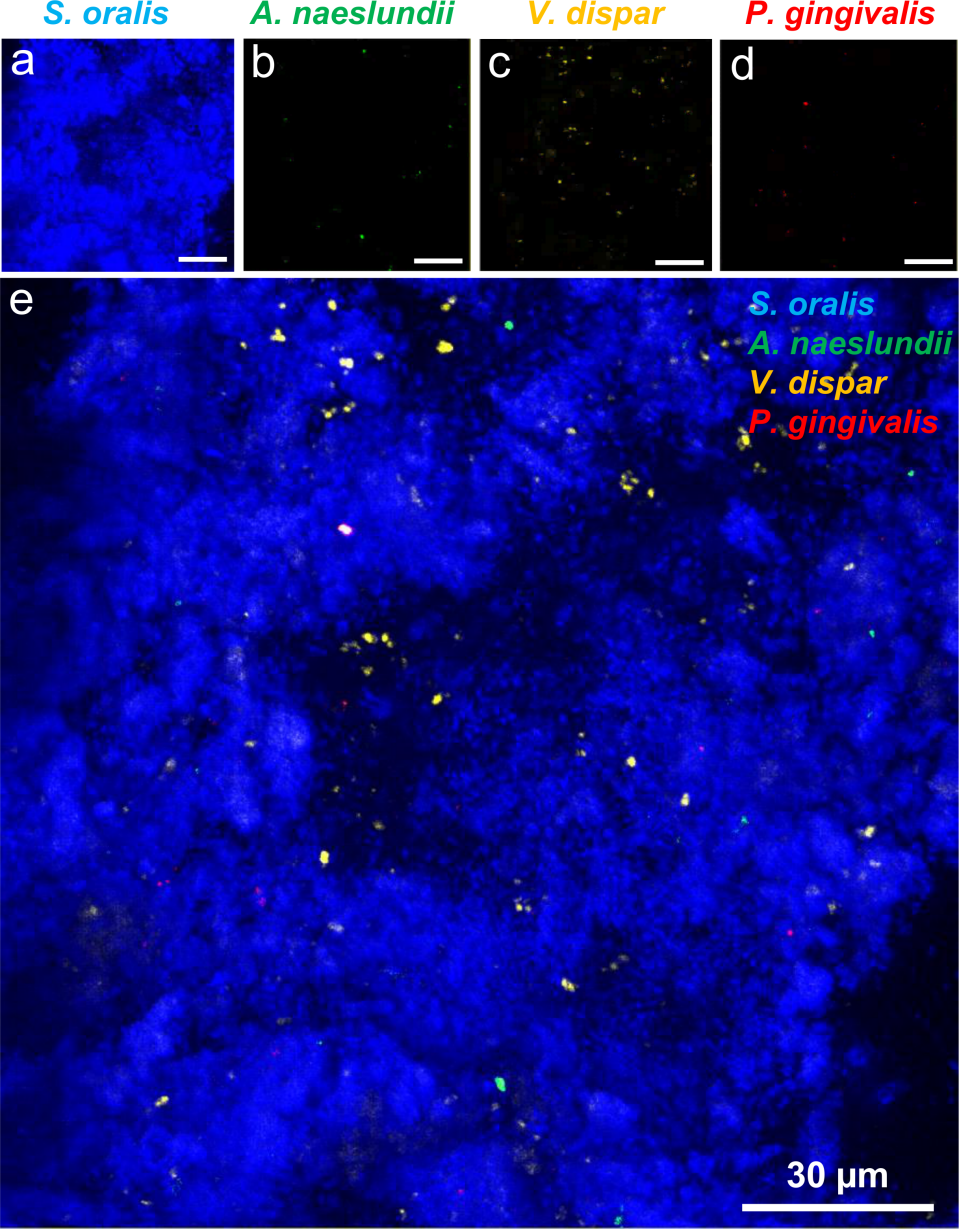
CLSM image of the FISH-stained 24 h four species biofilm on titanium specimen. Individual images with a z-step size of 2 μm of the 24 h four-species biofilm stained with species-specific 16S rRNA FISH probes for *S*. *oralis* (MIT-588-Alexa-405; blue), *A*. *naeslundii* (ANA-103-Alexa-488; green), *V*. *dispar* (VEI-217-Alexa-568; yellow) and *P*. *gingivalis* (POGI-Alexa-647; red) were overlaid to one image. (a)–(d) shows the individual color channels for the four individual species, (e) shows the overlay of the four color channels. Scale bars: 30μm.

FISH was carried out as an additional method to verify that each of the four species was included in the multispecies biofilm. The biofilms were stained with specific FISH-probes against the four individual species and the acquisition of 3D stacks of the biofilms was performed by CLSM. FISH revealed that each of the four species could be detected inside the biofilm after 24 hours of co-cultivation (Fig 4). As this method was only applied qualitatively, the amounts of the individual species were not determined.

## Discussion

Implant-associated infections still pose a severe problem in modern implant medicine. Novel approaches to combatting this problem necessitate reliable test systems for the analysis of oral biofilm formation on medical implant materials. In the present study, a protocol was developed to cultivate a four species oral biofilm model on titanium specimens in a flow chamber system. The reproducibility of multispecies biofilm formation on this common implant material was analyzed by means of fluorescent staining, CLSM, PMA-qRT-PCR and FISH.

Previously published flow chamber systems for the observation of oral biofilm formation have employed a variety of culture conditions. There are systems that use saliva as sole nutrient source [13,33,43], whereas others combine saliva, serum, simulated body fluid and/or nutrient broth [32,41,44,48] or solely use nutrient broth [36,42] to grow bacteria. The use of pooled saliva from human volunteers allows reliable biological analysis of bacterial interactions and mechanisms of biofilm formation [13,43]. However, it hinders the development of a highly standardized test system for intermediate to high sample numbers, due to the limited availability of closely defined saliva from an individual volunteer for a given set of experiments. A solution could be the use of artificial saliva. Nevertheless, not all bacteria are able to use saliva as sole nutrient source (e.g. *Streptococcus* spp.) or at least need specific co-aggregation partners for successful nutrient acquisition [13]. In this study, we decided to use solely commercial available, standardized nutrient broth for bacterial cultivation, as has been already successfully applied in the previous studies [36,40]. Besides nutrient source, the inoculation of bacteria to the flow system may include sequential inoculation of the individual species [41–43] or pre-mixing for inoculation [43,48]. In this study, bacterial species were pre-mixed to enhance comparability with the previous results with statically cultivated multispecies biofilms. This would also facilitate handling if the system is used in future to test innovative implant materials. Essential for cultivation in flow chamber systems is also the chosen flow rate. Saliva flow in the human mouth varies greatly from 0.1 ml/min to 7 ml/min [53]. In order to promote biofilm formation, a flow rate of 0.1 ml/min was chosen in this study, which is described as natural saliva flow in the hibernation mode [36,54,55]. The results of this study support the conclusion that the chosen cultivation conditions allowed reproducible growth of a four species biofilm in the flow chamber system.

Bacterial growth in the bioreactor feeding the flow chamber system was monitored by inline OD_600_ measurement and PMA-qRT-PCR. The resulting time/OD_600_ curve was highly reproducible and in accordance with typical bacterial growth curves. The distribution of viable species in the inoculum obtained by PMA-qRT-PCR was also highly reproducible between the different biological replicates. Comparison with the counted CFU/ml clearly shows that the two methods give different species distribution. The CFU method takes into account all cells which are able to grow, but a colony forming unit does not necessarily correspond to a single cell [56]. In contrast, viable cells detected by PMA-qRT-PCR are those with an intact membrane, as PMA treatment blocks DNA amplification from cells with disrupted membrane (dead) cells [40,49]. Therefore, the results of the two methods may differ. After 4 h of cultivation, the proportion of *S*. *oralis* increased in comparison to the other species, which indicated that *S*. *oralis* may be mainly responsible for the observed exponential growth. In the subsequent cultivation, the total numbers of *A*. *naeslundii* and *V*. *dispar* cells also increased. With increasing cultivation time, there appears to be greater variation in species abundance between the different biological replicates, especially for *V*. *dispar* and *P*. *gingivalis*. However, viable cells of all four species could be detected at all time points in all experiments. Therefore, co-cultivation of the four species in the bioreactor was achieved.

The biofilm that developed on titanium in the flow chambers was quantified by fluorescent staining and CLSM. Microscopy revealed that a multilayered biofilm formed on all titanium specimens. Our results further show that the biofilm volume was equivalent between the different biological replicates. The four-species biofilm volume was slightly lower than the volume of *S*. *oralis* grown as monospecies biofilm in the same flow chamber system [36], but considerably greater than the volume of the four-species biofilm grown statically in a multi-well plate [40]. The reduced growth compared to the monospecies biofilm can probably be attributed to interaction of *S*. *oralis* with other species, but also to the different nutrient broth used, which was supplemented with sucrose for monospecies cultivation. One reason that there was greater growth than in the static four-species cultivation may be the effect of different surface conditions (glass and titanium), but it would be more plausible to explain the findings with the better nutrient supply in the flow system. Live/dead staining additionally showed that all biofilms were mainly composed of vital cells. This is in line with the results of the statically grown four-species biofilm [40] and also with other oral multi-species biofilms grown in flow chamber systems [42,48]. The live/dead proportion differs between the biological replicates. Even if the system was always kept under the same conditions, e.g. nutrients and temperature, small changes in the environment may probably trigger a chain reaction in the complex process of multispecies biofilm formation in a flow system, resulting in small differences in cell viability. Nevertheless, this did not substantially influence biofilm formation in the flow chamber system.

The species abundance within the four-species biofilm was analyzed using PMA-qRT-PCR and FISH. Both methods showed that each of the four species formed a (viable) proportion of the overall biofilm biomass. While PMA-qRT-PCR enabled quantification of the distribution of individual species, FISH was only used for qualitative assessment. This indicated that *S*. *oralis* was the dominant species within the biofilm, whereas *V*. *dispar*, *A*. *naeslundii* and *P*. *gingivalis* were detected in considerably lower abundance. Since FISH staining required removal of titanium specimens from the flow chambers, the resulting hydrodynamic shear forces may have removed parts of the biofilm and thus may have biased FISH staining results.

Quantification via PMA-qRT-PCR revealed that *S*. *oralis* was always the dominant species and *P*. *gingivalis* was constantly at the lowest abundance in all three biological replicates. In one biological replicate (3.), *V*. *dispar* was the second and *A*. *naeslundii* the third most abundant species (Fig 3). That the quantitative composition is in the decreasing order *S*. *oralis*, *V*. *dispar*, *A*. *naeslundii* and *P*. *gingivalis*, exactly reflects the situation in our statically grown four-species biofilm [40]. In the other two biological replicates, the total abundance of *A*. *naeslundii* and *V*. *dispar* was interchanged—*A*. *naeslundii* was the second and *V*. *dispar* the third most frequent species. Despite these fluctuations, the counts of viable cells per ng DNA of *A*. *naeslundii* and *P*. *gingivalis* were equivalent within the three independent biological replicates. Moreover, the cell numbers of *S*. *oralis* and *V*. *dispar* were statistically equivalent within the first two biological replicates. As the cell numbers of *A*. *naeslundii* were equivalent through all independent biological replicates, the variations in the distribution of *A*. *naeslundii* and *V*. *dispar* were caused by changes in *V*. *dispar*. *V*. *dispar* indeed exhibited slightly higher standard deviations than the other species in the statically grown biofilms in multiwell-plates after 48 hours [40], which shows that the fluctuations of *V*. *dispar* were already evident in the static system. In addition, the volume of 1.8 liters in the current flow chamber system is obviously larger than the value of 150 μL in the previous 96-well plates, which suggests that a fluctuation is further enhanced by the greater volume. As already mentioned, the fluctuations of the *V*. *dispar* cell numbers were already evident after 4 hours of planktonic growth in the bioreactor, which is evident in Fig 1 as a higher standard deviation after 4 and 24 hours of planktonic growth. These fluctuations in the bioreactor also led to variations in the species distribution within the biofilm (Fig 3).

Foster and Kolenbrander [43] developed a multispecies biofilm model (*S*. *gordonii*, *A*. *naeslundii*, *V*. *atypica* and *F*. *nucleatum*) for basic biofilm research, in saliva pre-conditioned flow cells with saliva as sole nutrient and analyzed the influence of sequential (= each bacterial strain independently in a serial order) versus simultaneous (= with co-aggregates of mixed species) inoculation on subsequent biofilm composition and architecture. They could demonstrate—*inter alia*—that the amounts of *A*. *naeslundii* and *V*. *atypica* in biofilms inoculated as co-aggregates of mixed species were significantly higher than sequentially inoculated biofilms. They assumed that co-aggregations in planktonic precultures had an impact on the composition of the future multispecies biofilm [43]. In our study, we mixed the individual species and started the flow right after adding the four-species mixture to the bioreactor. In the following 24 hours, the bacteria had also the chance to co-aggregate in the bioreactor before forming a biofilm on the titanium specimens, which may also be a further reason for the slight fluctuations. Nevertheless, the distributions of the four species in the biofilms built up in the flow chambers were very similar to the ratios in our statically grown four-species biofilm model in 96-well plates. Both are in turn very similar to the *in vitro* biofilm model developed by Foster and Kolenbrander, which contained a related but also slightly fluctuating distribution of streptococci, actinomyces and veillonella [43]. The results of *in vivo* biofilm studies with enamel chips [12] were very similar to our 24 hour four species biofilm model, both statically [40] and dynamically, not just with respect to the proportion of streptococci [10,12], but also the distribution of veillonella (10%) and actinomyces (up to 7.8%).

The composition of the developed multispecies community represents a commensal oral biofilm, which is initially adhering on implant materials and thereby target to antibacterial surfaces. Commensal biofilms are mainly composed of initial colonizers, like streptococci, actinomyces and to a lower extend veillonellae, with streptococci accounting up to 80% of the biofilm [14,57–59]. Oral pathogens may already be present in the commensal biofilm, but to considerably lower amounts [57]. Their increase requires bridging organisms like *Fusobacterium nucleatum* [12,14,57]. These exhibit the required co-aggregation sites and can form metabolic relationships, for example in neutralizing the acidic pH produced by *S*. *oralis*, which is unfavorable for *P*. *gingivalis* [12,13,40]. Future experiments could address, if the addition of *F*. *nucleatum* could initiate a pathogenic shift of the here developed oral biofilm and increase the amount of *P*. *gingivalis*.

In conclusion, we successfully developed a system for the cultivation of a four-species oral biofilm model under flow conditions on titanium surfaces and demonstrated its reliability. With this new “Hanoverian Oral Multispecies Biofilm Implant Flow Chamber” (HOBIC) model, we provide an *in vitro* test system for biofilm formation on dental implants that more closely simulates the natural oral situation than monospecies biofilms or statically conducted experiments. In further studies, this system could be used, for example, to analyze the influence of nutrition and stress (pH, temperature, shear force) on oral biofilm formation with respect to biofilm volume, live/dead- and individual species distribution, as well as for application-oriented issues, such as the antimicrobial effects of promising compounds or putative new implant materials.

## Supporting information

S1 Table Primer pairs used in qRT-PCR to classify the different bacterial species. (DOCX)

S2 Table Reaction components for qRT-PCR. (DOCX)

S3 Table Thermal cycler conditions for qRT-PCR. (DOCX)

S4 Table Genome sizes, consulted accession numbers and the calculated genome weight used for individual cell count determination. (DOCX)

S5 Table Species-specific 16S rRNA probes for FISH. (DOCX)
