# Supplementary material for: Development and characterization of an oral multispecies biofilm implant flow chamber model
Source: PLoS One. 2018 May 17;13(5):e0196967. doi: 10.1371/journal.pone.0196967 (PMC5957423; doi:10.1371/journal.pone.0196967)
Supplement: S1 Table — (DOCX) [file pone.0196967.s001.docx]

**S1 Table. Primer pairs used in qRT-PCR to classify the different bacterial species.**

| **Species** | **Primer pairs** | **Annealing temp. [°C]** | **Gene** | **Expected size [bp]** | **Ref.** |
| --- | --- | --- | --- | --- | --- |
| *S. oralis* | F: 5’ - TCC CGG TCA GCA AAC TCC AGC C - 3’  R: 5’ - GCA ACC TTT GGA TTT GCA AC - 3’ | 58 | *gtfR* | 374 | [1, 2] |
| *A. naeslundii* | F: 5’ - CAA CGT CGA GGA GAT CCA GG - 3’  R: 5’ - TAT TGA GGA CCA CCT TGG CG - 3’ | 58 | *gyrA* | 215 | [2] |
| *V. dispar* | F: 5’ - TGG AGC AAA CCC GAG AAA CA - 3’  R: 5’ - TTC ACC GCA GTA TGC TGA CC - 3’ | 58 | *16S rRNA* | 104 | [2] |
| *P. gingivalis* | F: 5’ - AGG CAG CTT GCC ATA CTG CG - 3’  R: 5’ - ACT GTT AGC AAC TAC CGA TGT - 3’ | 56 | *16S rRNA* | 405 | [2, 3] |

**References**

1. Hoshino T, Kawaguchi M, Shimizu N, Hoshino N, Ooshima T, Fujiwara T. PCR detection and identification of oral streptococci in saliva samples using gtf genes. Diagn Microbiol Infect Dis. 48. United States2004. p. 195-9.

2. Kommerein N, Stumpp SN, Musken M, Ehlert N, Winkel A, Haussler S, et al. An oral multispecies biofilm model for high content screening applications. PLoS One. 2017;12(3):e0173973. Epub 2017/03/16. doi: 10.1371/journal.pone.0173973. PubMed PMID: 28296966.

3. Ashimoto A, Chen C, Bakker I, Slots J. Polymerase chain reaction detection of 8 putative periodontal pathogens in subgingival plaque of gingivitis and advanced periodontitis lesions. Oral Microbiol Immunol. 1996;11(4):266-73. Epub 1996/08/01. PubMed PMID: 9002880.
