# Supplementary material for: Development and characterization of an oral multispecies biofilm implant flow chamber model
Source: PLoS One. 2018 May 17;13(5):e0196967. doi: 10.1371/journal.pone.0196967 (PMC5957423; doi:10.1371/journal.pone.0196967)
Supplement: S2 Table — (DOCX) [file pone.0196967.s002.docx]

**S2 Table.**  **Reaction components for qRT-PCR.**

| **Component** | **Volume** | **Final Concentration** |
| --- | --- | --- |
| 2x Mastermix iQ^™^ SYBR^®^ Green Supermix  (Bio-Rad, Hercules, California, USA) | 12.5 µl | 1x |
| Forward Primer | 0.5 µl | 0.2 µM |
| Reverse Primer | 0.5 µl | 0.2 µM |
| Water, PCR grade  (Roche Life Science, Penzberg, Germany) | variable | - |
| Template DNA | variable (1-40 ng) | 40 pg – 1.6 ng |
| **Total Volume** | **25 µl** |  |
