# Supplementary material for: Development and characterization of an oral multispecies biofilm implant flow chamber model
Source: PLoS One. 2018 May 17;13(5):e0196967. doi: 10.1371/journal.pone.0196967 (PMC5957423; doi:10.1371/journal.pone.0196967)
Supplement: S3 Table — (DOCX) [file pone.0196967.s003.docx]

**S3 Table.**  **Thermal cycler conditions for qRT-PCR.**

| **Step** | **Temperature [°C]** | **Time [sec]** | **Cycles** |
| --- | --- | --- | --- |
| Pre-denaturation | 95 | 180 | 1x |
| Denaturation | 95 | 10 |  |
| Annealing | individual (see S1 Table) | 20 | 40x |
| Extension | 72 | 20 |  |
| Melting curve analysis | 60 | 6 | 150x |
