# Supplementary material for: Development and characterization of an oral multispecies biofilm implant flow chamber model
Source: PLoS One. 2018 May 17;13(5):e0196967. doi: 10.1371/journal.pone.0196967 (PMC5957423; doi:10.1371/journal.pone.0196967)
Supplement: S4 Table — (DOCX) [file pone.0196967.s004.docx]

**S4 Table.** **Genome sizes, consulted accession numbers and the calculated genome weight used for individual cell count determination.**

| **Species** | **Accession number** | **Genome size [bp]** | **Genome weight [ng]** |
| --- | --- | --- | --- |
| *S. oralis* | NC_015291.1 | 1.96E+06 | 2.15E-06 |
| *A. naeslundii* | ALJK00000000.1 | 3.04E+06 | 3.33E-06 |
| *V. dispar* | NZ_ACIK00000000.2 | 2.11E+06 | 2.32E-06 |
| *P. gingivalis* | NC_015571.1 | 2.34E+06 | 2.57E-06 |
