# Supplementary material for: Development and characterization of an oral multispecies biofilm implant flow chamber model
Source: PLoS One. 2018 May 17;13(5):e0196967. doi: 10.1371/journal.pone.0196967 (PMC5957423; doi:10.1371/journal.pone.0196967)
Supplement: S5 Table — (DOCX) [file pone.0196967.s005.docx]

**S5 Table.**  **Species-specific 16S rRNA probes for FISH.**

| **Probe** | **Species** | **Probe sequence [5’-3’]** | **Ref.** | **Label** |
| --- | --- | --- | --- | --- |
| MIT588 | *S. oralis* | 5’ - ACA GCC TTT AAC TTC AGA CTT ATC TAA- 3’ | (1, 2) | ALEXA Fluor^®^405 |
| ANA103 | *A. naeslundii* | 5’ - CGG TTA TCC AGA AGA AGG GG- 3’ | (1, 2) | ALEXA Fluor^®^488 |
| VEI217 | *V. dispar* | 5’ - AAT CCC CTC CTT CAG TGA- 3’ | (1-3) | ALEXA Fluor^®^568 |
| POGI | *P. gingivalis* | 5’ - CAA TAC TCG TAT CGC CCG TTA TTC- 3’ | (2, 4) | ALEXA Fluor^®^647 |

**References**

1. Thurnheer T, Gmur R, Guggenheim B. 2004. Multiplex FISH analysis of a six-species bacterial biofilm, p 37-47, J Microbiol Methods, vol 56, Netherlands.

2. Kommerein N, Stumpp SN, Musken M, Ehlert N, Winkel A, Haussler S, Behrens P, Buettner FF, Stiesch M. 2017. An oral multispecies biofilm model for high content screening applications. PLoS One 12:e0173973.

3. Paster BJ, Bartoszyk IM, Dewhirst FE. 1998. Identification of oral streptococci using PCR-based, reverse-capture, checkerboard hybridization | SpringerLink. Methods Cell Sci 20:223 – 231.

4. Sunde PT, Olsen I, Göbel UB, Theegarten D, Winter S, Debelian GJ, Tronstad L, Moter A. 2003. Fluorescence in situ hybridization (FISH) for direct visualization of bacteria in periapical lesions of asymptomatic root-filled teeth.
